# Tumors of the salivary gland in Mexicans. A retrospective study of 360 cases

**DOI:** 10.4317/medoral.17434

**Published:** 2011-12-06

**Authors:** Claudia P. Mejía-Velázquez, Marco A. Durán-Padilla, Erick Gómez-Apo, Daniel Quezada-Rivera, Luis A. Gaitán-Cepeda

**Affiliations:** 1 Professor, Dental School, National Autonomous University of Mexico, México; 2 Chief of the surgical pathology service, General Hospital of México, México city, México; 3 2nd year neuropathology resident, General Hospital of México, México city, México; 4 Chief of the Histopathologic Diagnosis Service, Graduate and Research Division, Dental School, National Autonomous University of Mexico, México; 5 Clinical and Experimental Pathology Laboratory, Graduate and Research Division, Dental School, National Autonomous University of Mexico, México

## Abstract

Objective: To establish distribution frequency and demographic characteristics of salivary gland tumours (SGT) i6n order to identify possible risk profiles. Design of study: The present report constitutes an eight year retrospective study (January 2000-August 2007). The archives of the Clinical and Experimental Pathology Laboratory (Graduate and Research Division, Dental School, National Autonomous University of Mexico) as well as archives of the Surgical Pathology Service (General Hospital, Mexico City) were subject to revision in order to select all cases where SGT tumour diagnoses were emitted. Age and gender of patients as well as SGT topography were obtained from medical records. Selected cases were classified according to location of the lesion, histological lineage and biological behaviour. Results: 360 cases of SGT were included, 227 (67%) cases were benign tumours, while 83 cases (23%) were malignant tumours. SGT were most frequent in women with ages ranging from their 3rd to 5th decades of life. 275 tumours were located in major salivary glands, 78.9% of them were identified in the parotid gland. The most frequent location of tumours arising from minor salivary glands (33 cases, 38%) was found in the palatine glands. Tumours of epithelial lineage were the predominant histological type. The most frequent benign tumours were pleomorphic adenomas (86.1%) and papillary cystadenoma lymphomatosum (7.3%). The most frequent malignant tumours were adenoid cystic carcinomas (25%) and mucoepidermoid carcinomas (23.6%) Conclusions: Salivary gland tumours in Mexican population appear principally in major salivary glands of women in their 3rd to 5th decade of life.

** Key words:** Salivary glands tumours, epithelial tumours, pleomorphic adenoma, papillary cistadenoma lymphomatosum, adenoid cystic carcinoma, mucoepidermoid carcinoma.

## Introduction

Salivary gland tumours (SGT) are uncommon entities that amount to 3% to 10% of all head and neck neoplasms (1). This low incidence could be related to racial and geographical factors. The age-adjusted annual incidence is 4.7 % for benign SGT cases and 0.9 % for malignant ones (2).

SGT have preference for women in their 3rd to 5th decades of life (3-12). 50% of all SGT are benign, more than half of these arise from major salivary glands, 64 to 80% of them in the parotid gland (4). When defining histological type, the most frequent variety are the pleomorphic adenomas (PA) for benign tumours, and mucoepidermoid carcinomas for malignant ones (4-7, 10-12).Tumours that originate in minor salivary glands represent 10 to 25% of all SGT. The majority of these tumours are malignant (50-60%), and mucoepidermoid carcinoma and cystic adenoid carcinoma are the most frequent (1,13-19).

Since scientific information (1-23) related to this issue is scarce, topographical and frequency distribution as well as demographic characteristics of SGT are unknown for Latin American in general as well as in particular for the Mexican population. This research aims at contributing to the identification of risk profiles through the establishment of demographic characteristics of patients afflicted with SGT.

## Material and Methods

Records dated from January 2000 to August 2007 of the Laboratory of Clinical and Experimental Pathology, Graduate and Research Division, Dental School, National Autonomous University of Mexico and of the Surgical Pathology Service of the General Hospital of Mexico City were examined to identify and select all cases of diagnosed salivary gland (major or minor) tumours. Age and gender of patients and location of lesions were obtained from medical records. Cases with sufficient biological material embedded in paraffin or histological slides stained with Hematoxylin and Eosin technique were reviewed by two pathologists (CPMV/MADP) to confirm diagnosis, or to re-diagnose and classify the cases following criteria proposed by the World Health Organization in 2005 for salivary gland tumours. Additionally, cases were grouped according to their behaviour (benign or malignant), histogenesis (epithelial, lymphoid and mesenchymal) and topography/location (major or minor salivary glands). 

An ex professo database was achieved using the software programme SPSS 16.0®. Data on frequency of tumours with respect to age and gender of patients, location, and histogenesis were obtained.

## Results

**Figure 1:**
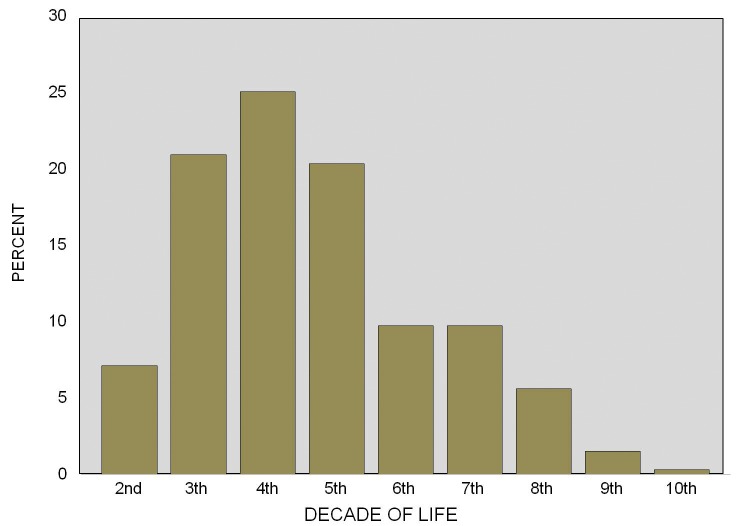
Age distribution of salivary gland tumors in a mexican population.

169,051 files from the Surgery Pathology Service of the General Hospital of Mexico City were examined. Out of these, 471 cases with diagnoses of SGT were identified. From these 471 identified cases, 152 were discarded due to lack of sufficient biological material or histological slides stained with Hematoxylin and Eosine technique, or because the diagnosis did not correspond with that of a primary tumour of the salivary glands. 319 cases were then included in the present study. Additionally, 6,548 files from the records of Laboratory of Clinical and Experimental Pathology, Graduate and Research Division, School of Dentistry, National Autonomous University of Mexico were examined. Out of these, 53 cases with SGT diagnosis were identified and selected. 12 cases were discarded for reasons similar to the aforementioned, and 41 cases of SGT were included. From this point onwards, and for logistics reasons, both case series were combined to form a single database of 360 cases. From the total sample (n=360), 230 cases were women (average age 41.9 years; standard deviation [SD] ±16.2) and 130 were men (average age 42.1 (SD ±17.4). Age range of all samples was 11-93 years with an average of 42 years (SD ±16.6). Figure 1 shows age distribution of all cases.

Out of these 360 cases, 76.3% were located in major salivary glands while 64 cases (17.7%) were found in minor salivary glands. In 21 cases, location was impossible to establish. The most common location of the major SGT was the parotid gland (78%) followed by the submandibular gland with 20.7%. Only one case was identified in the sublingual gland. With respect to minor SGT, the palatine gland was the most frequent location, percentage being 51.5% (33 cases). The lip followed in location frequency with 10 cases, then the tongue with 5 cases, after this the oral mucosa, with 5 cases, and finally the retromolar area with 3 cases.  Table 1 shows the topographical distribution of the total sum of cases.

 Behaviour and histological lineage.

277 cases were benign tumours and 83 cases were malignant tumours. 274 benign tumours were of epithelial origin, while 3 were mesenchymal tumours. 72 malignant tumours were of epithelial origin and 11 cases were lymphoid.  Table 2shows total distribution of SGT with respect to their behaviour and histological lineage.

 -Epithelial tumours ( n= 346)

Epithelial tumours constituted 96.1% of the whole sample. These tumours showed predilection for patients in their third up to their fifth decade of life. 219 (63.2%) were women, with age average of 41.7 years (SD ±15.8), 127 were men, with age average of 41.9 years (SD ±17.5). In 261 cases tumours were located in major salivary glands, distributed as follows: 206 (78.9%) in the parotid gland, 54 (20.7%) in the submandibular gland, and only one (0.4%) in the sublingual gland. 64 cases were located in the minor salivary glands. Of these, the palate was found to be the most frequent location ( Table 3). 274 (79.1%) tumours were benign, with peak of incidence in patients in their fourth decade of life. 176 (64.2%) patients were women and 98 (35.7%) were men. In the 72 (20.8%) remaining cases (43 [59.7%] women; 29 [40.2%] men) the lesions were malignancies, patient age ranging from 17 to 85 years, with predominance of patients in their 5th decade of life.

 -Histological subtype

 -Benign Tumours

 Pleomorphic adenoma. Pleomorphic adenoma was the most common of the benign tumours, accounting for 236 cases. Mean age at diagnosis time was 39.9 years (SD ±15.3), with female predominance. The most frequent location were the major salivary glands, with 193 cases. The parotid gland was the most frequent site (76.7%), followed by the submandibular gland (22.8%). With respect to the minor salivary glands the palate (53.8%), lip (22.2%) and oral mucosa (8.3%) were the most frequent locations.

 Papillary Cystadenoma Lymphomatosum (PCL). The 20 cases of PCL represented 7.3% of all benign epithelial neoplasms. PCL showed an incidence in patients in their 5th and 7th decade. Male-female ratio was 2:1. The parotid gland was the most common site accounting for 80% of cases. 2 cases were found in the submandibular gland, and specific location of two other cases could not be obtained.

 Basal cell adenoma. 8 cases (2.9%) of basal cell adenoma were diagnosed. Mean age of patients was 46.5 years (SD ±16.6). The majority of patients were women (62.5%) with a peak of lesions in their 7th decade of life. Six cases were found in the parotid gland. Location of tumours in other cases was unobtainable.

 Others. Mioepitheliomas represented 1.5% of all tumours. Average age of patients was 40.75 years (range 22-74, female-male ratio 3:1). With respect to their topography, three cases were found in the palate and one in the parotid gland. Three cases of cystadenoma were identified. Mean age of patients was 40.6 years (SD ±23). All three cases were in the parotid gland of female patients. Oncocytomas represented 0.7% (2 cases) of all SGT. Both cases were located in the parotid gland, one case was found in a 35 year old woman and the other in a 59 year old man. There was only one case of ductal adenoma and it was located in the parotid gland of a 42 year old woman.

 -Malignant tumours

 Adenoid cystic carcinoma (ACC). ACC constituted 25% of all epithelial malignancies. ACC showed a peak of incidence in patients in their 5th decade of life, without gender predilection. Minor salivary glands were the most frequent site, specifically in the palate and maxillary sinus.

 Mucoepidermoid carcinoma. Mucoepidermoid carcinomas rated second in frequency of malignant lesions, accounting for 17 cases. Mucoepidermoid carcinoma was most frequent in women in their 5th decade of life. 64.7% of all mucoepidermoid carcinomas arose from minor salivary glands, the most frequent location being the palate, followed by lip, tongue, retromolar area and oral mucosa.

 Acinar cell carcinoma. 9 cases of acinar cell carcinoma were diagnosed, representing 12.5% of all malignant epithelial tumours. Acinar cell carcinoma was found in patients ranging from 20 to 59 years. A slight predominance of cases in males was observed. All cases examined were found in the parotid gland.

 Ex-adenoma pleomorphic carcinoma. This malignant tumour represents 9.7% of all malignancies. Ex adenoma pleomorphic carcinoma was most frequently found in patients in their 4th decade of life. 57.1% of these lesions were found in men, all of them in major salivary glands.

Salivary duct carcinoma. Only 5 cases of salivary duct carcinoma were identified. The tumours were mainly located in the parotid gland and appeared predominantly in male patients with ages ranging from 32 to 72 years.

 Lymphoid tumours (n=11)

11 (3.1% of the total SGT sample) lymphoid tumours were identified. These tumours appeared predominantly in females in the 22-72 age bracket (average age 53 years). Nine cases were found in the parotid gland and two in the submandibular gland. Lymphoma of mucous membrane associated lymphoid tissue (MALT) was the most frequent histological subtype (54.5% of all lymphomas), it appeared predominantly in women. All lymphomas were located in the parotid gland. The diffuse large cell B lymphoma constituted 27% of the sample. It showed predilection for women and to be located in the parotid gland. One was found in the submandibular gland. Two cases of follicular lymphoma were diagnosed, one in the parotid gland and one in the submandibular gland. Follicular lymphoma represented 18.2%.

 Mesenchymal tumours (n=3)

Only three mesenchymal tumours were identified. Mesenchymal tumours constituted 8 % of the whole sample. Two cases were hemangiomas, found in the submandibular gland and parotid gland of two women of 15 and 31 years, respectively. The remaining case was a schwannoma located in the parotid gland of a 20 year old woman.

**Table 1:**
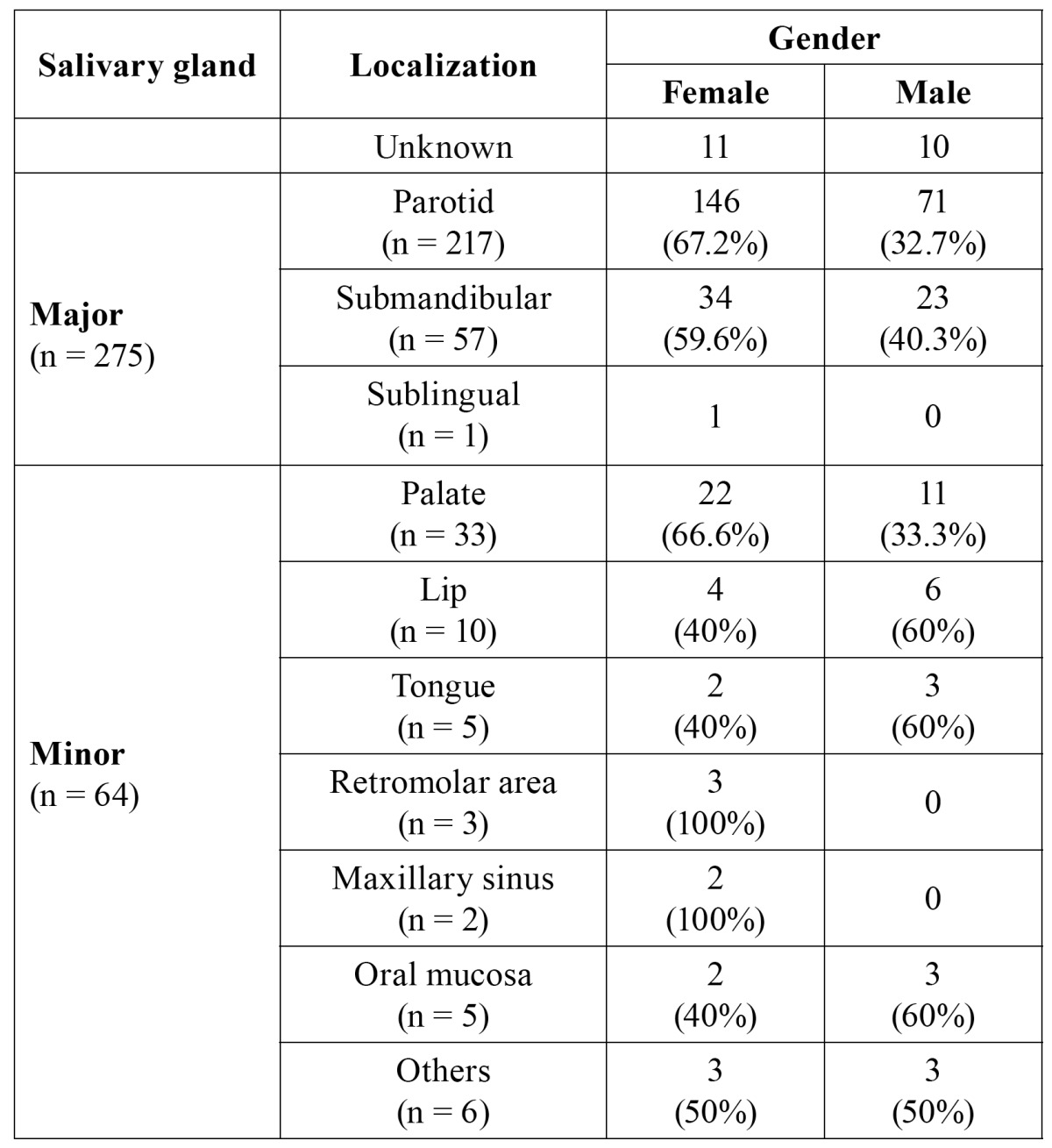
Gender distribution of major and minor salivary gland tumors.

**Table 2:**
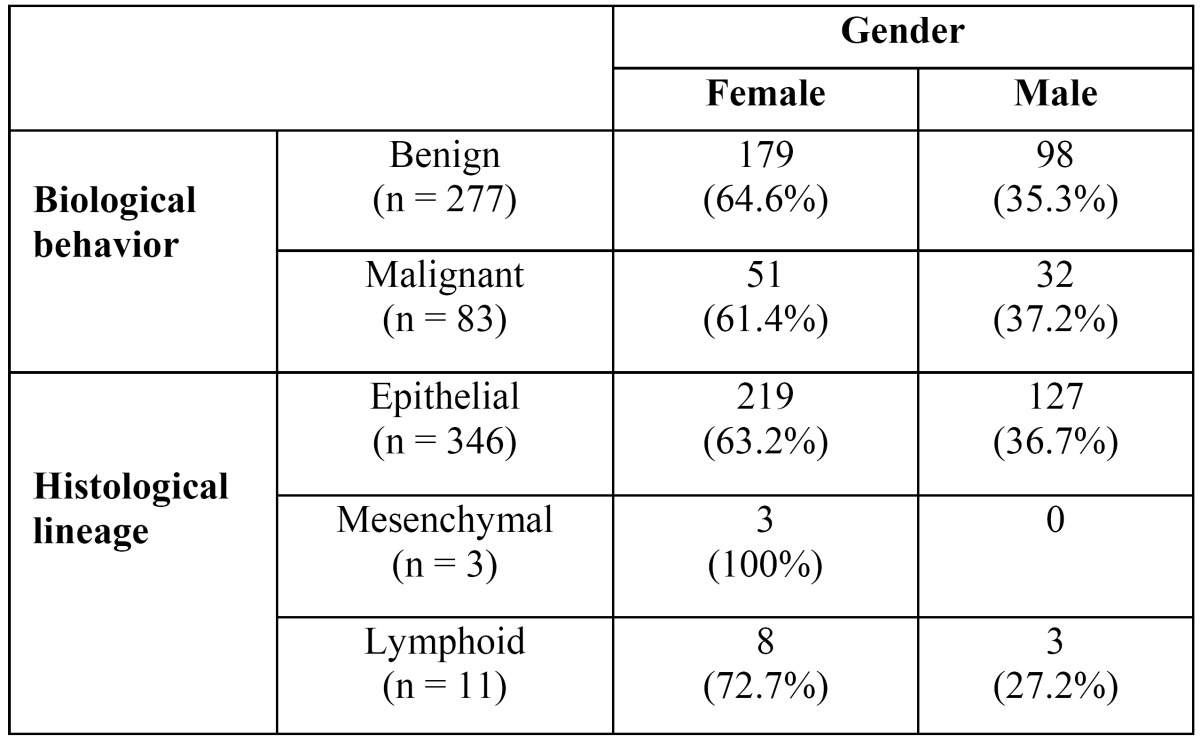
Gender distribution and biological behavior.

**Table 3:**
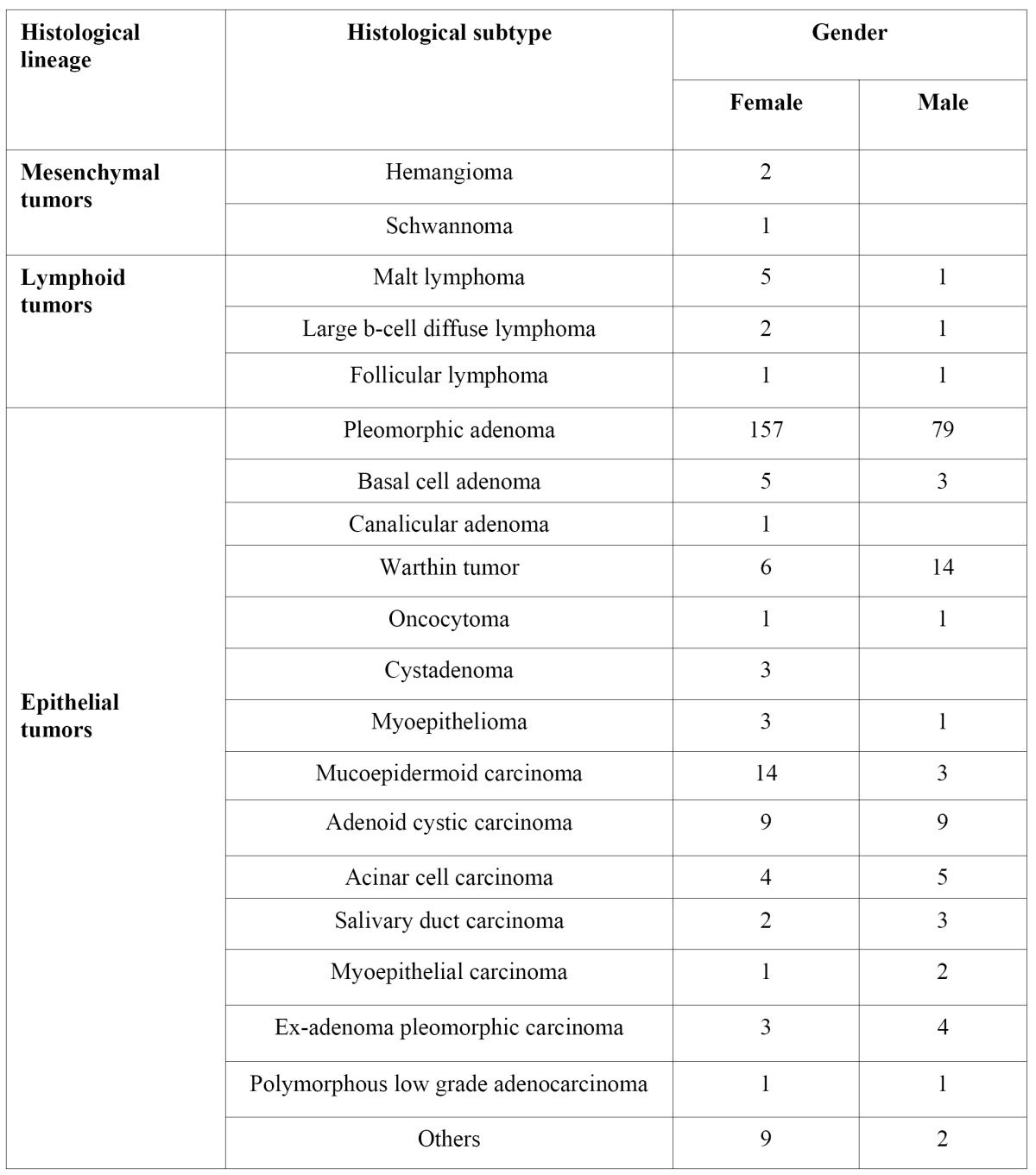
Gender distribution and histological subtype.

## Discussion

This paper describes demographic characteristics of 360 salivary glands tumours, including epithelial, mesenchymal and lymphoid tumours. Our results concur with previous reports with respect to age and gender of patients and location and histogenesis of tumours. Nevertheless, a previous report carried out in Mexican population (20), suggests that the preferred site of SGT is the minor salivary glands. Data gathered in the present research do not concur. A possible explanation for this apparent contradiction could reside in the fact that the archive revised by Ledesma-Montes and Garcés-Ortiz (20) and reviewed once more by the authors of the present study, includes only samples from head and neck services, where the majority of surgical specimens originate from intraoral surgeries. Therefore, the surgical specimens of minor salivary glands are prevalent. This report includes samples gathered from a second level hospital with high national concentration of patients, which would then suggest a more accurate representation of the Mexican population.

In this report, PCL was the second most frequent benign epithelial neoplasia. Male:female ration was 1:2 These findings concur with the report of Ito et al (9). Recently, scientific literature has described an increase in female patients, shifting the male:female ratio to 1:1 (2,11). Ito´s et al report (9) as well as the present one, both carried out in Latin American populations, disagree with the aforementioned suggestion. These apparent discrepancies could be related to smoking habits, which are the main etiological factor for this particular neoplasia. Latin America is experiencing a recent increase in women smokers, it could then be suggested that the next decade will see a shift in trend. It is interesting to point out that two PCL cases were diagnosed in the submandibular gland, since it is known that this neoplasia is almost exclusive of the parotid gland. (2,4,9-11). In both cases, the medical file did not provide sufficient clinical data to rule out an extension of primary tumour from the parotid gland. Cases of PLC arising from the minor salivary glands have been reported (19,20).

In this study, the highest prevalence of malignant epithelial tumours was observed in patients in the 5th decade of life which concurs with the report of Ledesma-Montes and Garcés Ortiz (20). It should be noted that both studies show lesser prevalence of malignant epithelial tumours than other studies described in scientific literature. A peak of prevalence of these lesions in patients in their 6th-7th decade of life has been reported (1,5,9,10,13,17,19). A fact to be noted is that mucoepidermoid carcinoma showed predilection for minor salivary glands, which disagrees with other reports in the scientific literature (4-7,9,11,12). In theses cases, women were strong favourites, (female:male ratio 7:1) To this date, no explanation has been found to justify this ratio. Al Khaleeb et al (11) informed of a similar association.

The greater part of published SGT series are restricted to epithelial tumours. Few reports included non-lymphoid mesenchymal tumours (9-11).This report studied three mesenchymal cases, which corresponds to 0.8% of the total sample. This figure is slightly under the reported ratio of 1 to 2% (9-11). The cases studied in this report were two hemangiomas and one schwannoma. The latter is considered one of the most frequent tumours along with lipoma, neurofibroma and lymphangioma (9-11,24-26). With respect to lymphoid tumours, the most frequent histological kind encountered was the cell B non-Hodgkin lymphoma. All of them were located in major salivary glands, mainly in female patients. With respect to the histological subtype of lymphomas, and in accordance with other findings in scientific literature, we observed predominance of extraganglionar marginal zone lymphomas (2,27).

It can be concluded that the present study shows that in Mexican population salivary gland tumours can be found mainly in salivary glands of women in their 3rd to 5th decades of life. Benign epithelial tumours were the most frequent. Malignant tumours were found in younger age brackets than other cases reported in scientific literature. This suggests a change in the demographic profile of salivary gland adenocarcinomas in Mexican population. This suggestion must be corroborated by other research groups. If this confirmation takes place, a research protocol should be devised focusing on the identification of the possible factor which could explain the demographical differences proposed here, including racial or geographical factors.

## References

[1] Eveson JW, Cawson RA (1985). Tumours of the minor (oropharyngeal) salivary glands: a demographic study of 336 cases. J Oral Pathol.

[2] Pinkston JA, Cole P (1999). Incidence rates of salivary gland tumors: results from a population-based study. Otolaryngol Head Neck Surg.

[3] Foote FW, Frazell EL (1953). Tumors of the major salivary glands. Cancer.

[4] Eneroth CM (1971). Salivary gland tumors in the parotid gland, submandibular gland and the palate region. Cancer.

[5] Main JH, Orr JA, Mc Gurk FM, Mc Comb RJ, Mock D (1976). Salivary gland tumors: review of 643 cases. J Oral Pathol.

[6] Spiro RH (1986). Salivary neplasms: Overview of a 35-year experience with 2, 807 patients. Head & Neck Surg.

[7] Chidzonga MM, Lopez Perez VM, Portilla-Alvarez AL (1995). Salivary gland tumours in Zimbabwe: report of 282 cases. Int J Oral Maxillofac Surg.

[8] Perez DE, Pires FR, Alves F de A, Almeida OP, Kowalski LP (2005). Sublingual salivary gland tumors: Clinicopathologic study of six cases. Oral Surg Oral Med Oral Pathol Oral Radiol Endod.

[9] Ito FA, Ito K, Vargas PA, de Almeida OP, Lopes MA (2005). Salivary gland tumors in a Brazilian population: a retrospective study of 496 cases. Int J Oral Maxillofac Surg.

[10] Otoh EC, Johnson NW, Olasoji H, Danfillo IS, Adeleke OA (2005). Salivary gland tumors in neoplasms in Maiduguri, north-eastern Nigeria. Oral Dis.

[11] Al-Khateeb TH, Ababneh KT (2007). Salivary tumors in north Jordanians: a descriptive study. Oral Surg Oral Med Oral Pathol Oral Radiol Endod.

[12] Ansari MH (2007). Salivary gland tumors in an Iranian population: a retrospective study of 130 cases. J Oral Maxillofac Surg.

[13] Waldron CA, el-Mofty SK, Gnepp DR (1988). Tumors of the intraoral minor salivary glands: a demographic and histologic study of 426 cases. Oral Surg Oral Med Oral Pathol.

[14] Neville BW, Damm DD, Weir JC, Fantasia JE (1988). Labial salivary gland tumors. Cancer.

[15] Jansisyanont P, Blanhaert RH Jr, Ord RA (2002). Intraoral minor salivay gland neoplasm: a single institution experience of 80 cases. Int J Oral Maxillofac Surg.

[16] Yih WY, Kratochvil FJ, Stewart JC (2005). Intraoral minor salivary gland neoplasms: review of 213cases. J Oral Maxillofac Surg.

[17] Toida M, Shimokawa K, Makita H, Kato K, Kobayashi A, Kusunoki Y et al (2005). Intraoral minor salivary gland tumors: a clinicopathological study of 82 cases. Int J Oral Maxillofac Surg.

[18] Jaber MA (2006). Intraoral minor salivary gland tumors: a review of 75 cases in a Libyan population. Int J Oral Maxillofac Surg.

[19] Wang D, Li Y, He H, Liu L, Wu L, He Z (2007). Intraoral minor salivary gland tumors in a Chinese population: a retrospective study on 737 cases. Oral Surg Oral Med Oral Pathol Oral Radiol Endod.

[20] Ledesma-Montes C, Garces-Ortiz M (2002). Salivary gland tumours in a Mexican sample A retrospective study. Med Oral.

[21] Takahashi H, Fujita S, Tsuda N, Tezuka F, Okabe H (1990). Intraoral minor salivary gland tumors: a demographic and histologic study of 200 cases. Tohoku J Exp Med.

[22] van der Wal JE, Snow GB, van der Waal I (1992). Histological reclassification of 101 intraoral salivary gland tumors (New WHO classification). J Clin Pathol.

[23] van der Wal JE, Carter RL, Klijanienko J, Micheau C, Rilke F, Seifert G (1993). Histological re-evaluation of 101 intraoral salivary gland tumors by an EORTC-study group. J Oral Pathol Med.

[24] Cho KJ, Ro JY, Choi J, Choi SH, Nam SY, Kim SY (2008). Mesenchymal neoplasms of the major salivary glands: clinicopathological features of 18 cases. Eur Arch Otorhinolaryngol.

[25] Takahama A JR, León JE, de Almeida OP, Kowalski L (2008). Nonlymphoid mesenchymal tumors of the parotid gland. Oral Oncol.

[26] Childers EL, Furlong MA, Fanburg-Smith JC (2002). Hemangioma of the salivary gland: a study of ten cases of a rarely biopsied/excised lesion. Ann Diagn Pathol.

[27] Roh JL, Huh J, Suh Ch (2008). Primary non-Hodgkin’s Lymphomas of the major salivary glands. J Surg Oncol.

